# Effect of Nutritional Determinants and TonB on the Natural Transformation of *Riemerella anatipestifer*

**DOI:** 10.3389/fmicb.2021.644868

**Published:** 2021-08-10

**Authors:** Li Zhang, Li Huang, Mi Huang, Mengying Wang, Dekang Zhu, Mingshu Wang, Renyong Jia, Shun Chen, Xinxin Zhao, Qiao Yang, Ying Wu, Shaqiu Zhang, Juan Huang, Xumin Ou, Sai Mao, Qun Gao, Bin Tian, Anchun Cheng, Mafeng Liu

**Affiliations:** ^1^Institute of Preventive Veterinary Medicine, Sichuan Agricultural University, Chengdu, China; ^2^Research Centre of Avian Disease, College of Veterinary Medicine of Sichuan Agricultural University, Chengdu, China; ^3^Key Laboratory of Animal Disease and Human Health of Sichuan Province, Chengdu, China

**Keywords:** *Riemerella anatipestifer*, natural transformation, peptone, phosphate, iron, tonB

## Abstract

*Riemerella anatipestifer* is a gram-negative bacterium that is the first naturally competent bacterium identified in the family *Flavobacteriaceae*. However, the determinants that influence the natural transformation and the underlying mechanism remain unknown. In this study, we evaluated the effects of various nutritional factors of the GCB medium [glucose, L-glutamine, vitamin B1, Fe (NO_3_)_3_, NaCl, phosphate, and peptone], on the natural transformation of *R. anatipestifer* ATCC 11845. Among the assayed nutrients, peptone and phosphate affected the natural transformation of *R. anatipestifer* ATCC 11845, and the transformation frequency was significantly decreased when phosphate or peptone was removed from the GCB medium. When the iron chelator 2,2′-dipyridyl (Dip) was added, the transformation frequency was decreased by approximately 100-fold and restored gradually when Fe (NO_3_)_3_ was added, suggesting that the natural transformation of *R. anatipestifer* ATCC 11845 requires iron. Given the importance of TonB in nutrient transportation, we further identified whether TonB is involved in the natural transformation of *R. anatipestifer* ATCC 11845. Mutation of *tonBA* or *tonBB*, but not *tbfA*, was shown to inhibit the natural transformation of *R. anatipestifer* ATCC 11845 in the GCB medium. In parallel, it was shown that the *tonBB* mutant, but not the *tonBA* mutant, decreased iron acquisition in the GCB medium. This result suggested that the *tonBB* mutant affects the natural transformation frequency due to the deficiency of iron utilization.

## Introduction

Natural transformation is the process by which bacteria take up and integrate exogenous DNA into their genomes (Seitz and Blokesch, 2013). Most gram-negative bacteria use the type IV pilus family (T4P) to retract DNA into the periplasm, where one strand is degraded and the other is internalized into the cytoplasm through the ComEC transmembrane channel. Then, the internalized single-stranded DNA is bound by DprA. With the participation of RecA and ComM, the translocated strand replaces a chromosomal strand by recombination (Dubnau and Blokesch, 2019).

Transformation promotes the generation of new genetic traits and helps bacteria to adapt to new environmental conditions (Croucher et al., 2011). Thus, environmental determinants are important factors affecting natural transformation. Natural transformation has been observed and thoroughly studied in a wide variety of bacterial species, including *Streptococcus pneumoniae* (*S. pneumoniae*) (Lefrancois et al., 1998; Johnsborg and Havarstein, 2009; Straume et al., 2015), *Neisseria* (Facius and Meyer, 1993; Hamilton and Dillard, 2006; Zhang et al., 2013), *Bacillus subtilis* (*B. subtilis*) (Kosovich and Prozorov, 1990; Brautigam et al., 1997; Le et al., 2017), *Haemophilus influenzae* (*H. influenzae*) (Redfield, 1993; Mell et al., 2011), *Vibrio cholerae* (*V. cholerae*) (Frischer et al., 1990; Watve et al., 2014; Dalia et al., 2017), and *Acinetobacter baylyi* (*A. baylyi*) (Vaneechoutte et al., 2006; Merod and Wuertz, 2014; Utnes et al., 2015; Hulter et al., 2017; Leong et al., 2017; Suarez et al., 2017; Nero et al., 2018; Mantilla-Calderon et al., 2019). Although most bacteria possess competence genes, numerous conditions or signals trigger competence and are often species specific (Seitz and Blokesch, 2013). The expression of competence genes is influenced by the growth phase, cell density, metabolic activity, nutritional stress, and DNA damage (Johnsborg et al., 2007). For example, in *S. pneumoniae* and *Helicobacter pylori*, competence is induced by antibiotic stress or DNA damage (Prudhomme et al., 2006; Dorer et al., 2010).

*Riemerella anatipestifer* is a gram-negative bacterium that infects ducks, geese, turkeys, and other birds, and in ducklings, it can lead to a large number of deaths, resulting in huge economic losses (Wang et al., 2017). Currently, at least 21 different *R. anatipestifer* serotypes have been identified, among which, no cross-protection occurs (Helfer and Helmboldt, 1977; Leavitt and Ayroud, 1997), making eradication of this bacterium difficult. Previously, *R. anatipestifer* was described as a constitutive naturally transformable bacterium, although the genes that encode the natural transformation machinery were not identified completely (Liu et al., 2017b). Until now, several *R. anatipestifer* strains have been sequenced, and a comparison of the strains has revealed that the genomes are diverse (Wang et al., 2014; Liu J. et al., 2019). The genetic diversity could be caused by mutations, genomic rearrangements, and foreign DNA from the surrounding region. Additionally, *R. anatipestifer* exhibits resistance to multiple classes of antibiotics, including aminoglycosides, cephalosporins, chloramphenicol, lincosamides, macrolides, and nalidixic acid (Gyuris et al., 2017; Huang et al., 2017; Zhang et al., 2017; Luo et al., 2018), but the source of these resistance genes remains largely unknown. Thus, natural transformation can be hypothesized to play an important role in genomic diversity, the dissemination of antibiotic resistance, and evolution.

In this study, to understand the effect of nutritional determinants on the natural transformation of *R. anatipestifer* ATCC 11845, we investigated the efficiency of natural transformation under different nutrient conditions and found that peptone, phosphate, and iron influenced the natural transformation of *R. anatipestifer* ATCC 11845. Since the TonB of gram-negative bacteria is important for nutrient transportation, we further investigated the effect of TonB on the natural transformation of *R. anatipestifer* ATCC11845. *R. anatipestifer* encode ExbB-ExbD-TonB (TonB1 system), ExbB-ExbD-ExbD-TonB (TonB2 system), and TonB family protein (TbfA) for nutrient utilization (Liao et al., 2015; Liu et al., 2016). Here, TonB of TonB1 system and TonB2 system are re-termed as TonBA and TonBB, respectively. We found that the deletion of the *tonBA* and *tonBB* genes decreased the natural transformation frequency of *R. anatipestifer* ATCC 11845.

## Materials and Methods

### Bacterial Strains, Plasmids, and Primers

The bacterial strains and plasmids used in this study are listed in Table 1. The primer sequences used in this study are listed in Table 2.

**TABLE 1:** Strains and plasmids used in this study.

*Escherichia coli* strains	Genotype	Source or reference
XL1-BLUE	F^–^ *supE44 hdsR17 recA1 endA1 gyrA46 thi relA1* lac^–^ F’ *proAB*^–^ *lacI*^*q*^ *lacZ*Δ*M15* Tn*10*, Tet^*R*^	Laboratory collection
DH5α	F^–^ Φ80lacZΔM15 Δ(*lacZYA-argF*) U169 *recA1 endA1 hsdR17* (rK–, mK +) *phoA supE44*λ*– thi-1 gyrA96 relA1*	Laboratory collection
S17-1	*hsdR17 recA1* RP4-2-*tet:*Mu-1kan:Tn7; Sm^*R*^	Miller and Mekalanos, 1988

***Riemerella anatipestifer* strains**	**Genotype or serotype**	**Source or reference**

*R. anatipestifer* ATCC 11845	RA ATCC 11845, Km^*R*^	Wang et al., 2012
*R. anatipestifer*ATCC 11845*ΔRA0C_1551*	RA ATCC 11845, *RA0C_1551* mutant, Erm^*R*^	Liu et al., 2017b
*R. anatipestifer* ATCC 11845 pLMF03	RA ATCC 11845, pLMF03, Cfx^*R*^	Liu et al., 2018
*R. anatipestifer* ATCC 1184*5ΔtonBA* pLMF03	RA ATCC 11845, *tonBA* mutant, pLMF03, Cfx^*R*^	This study
*R. anatipestifer* ATCC 1184*5ΔtonBA* pLMF03:*tonBA*	RA ATCC 11845, *tonBA* mutant, pLMF03:*tonBA*, Cfx^*R*^	This study
*R. anatipestifer* ATCC 11845*ΔtonBB* pLMF03	RA ATCC 11845, *tonBB* mutant, pLMF03, Cfx^*R*^	This study
*R. anatipestifer* ATCC 1184*5ΔtonBB* pLMF03:*tonBB*	RA ATCC 11845, *tonBB* mutant, pLMF03:*tonBB*, Cfx^*R*^	This study
*R. anatipestifer* ATCC 11845*ΔtbfA* pLMF03	RA ATCC 11845, *tbfA* mutant, pLMF03, Cfx^*R*^	This study
*R. anatipestifer* ATCC 1184*5ΔtbfA* pLMF03:*tbfA*	RA ATCC 11845, *tbfA* mutant, pLMF03:*tbfA*, Cfx^*R*^	This study
*R. anatipestifer* ATCC 11845*ΔtonBAΔtonBB* pLMF03	RA ATCC 11845, *tonBA* mutant, *tonBB* mutant, pLMF03, Cfx^*R*^	This study
*R. anatipestifer* ATCC 11845*ΔtonBAΔtonBB* pLMF03:*tonBA*	RA ATCC 11845, *tonBA* mutant, *tonBB* mutant, pLMF03:*tonBA*, Cfx^*R*^	This study
*R. anatipestifer* ATCC 11845*ΔtonBAΔtonBB* pLMF03:*tonBB*	RA ATCC 11845, *tonBA* mutant, *tonBB* mutant, pLMF03:*tonBB*, Cfx^*R*^	This study
*R. anatipestifer* ATCC 11845*ΔtonBAΔtonBBΔtbfA* pLMF03	RA ATCC 11845, *tonBA* mutant, *tonBB* mutant, *tbfA* mutant, pLMF03, Cfx^*R*^	This study
*R. anatipestifer* ATCC 1184*5ΔtonBAΔtonBBΔtbfA* pLMF03:*tonBA*	*R. anatipestifer* ATCC 1184*5, tonBA* mutant, *tonBB* mutant, *tbfA* mutant, pLMF03:*tonBA*, Cfx^*R*^	This study
*R. anatipestifer* ATCC 1184*5ΔtonBAΔtonBBΔtbfA* pLMF03:*tonBB*	*R. anatipestifer* ATCC 1184*5, tonBA* mutant, *tonBB* mutant, *tbfA* mutant, pLMF03:*tonBB*, Cfx^*R*^	This study
*R. anatipestifer* ATCC 1184*5ΔtonBAΔtonBBΔtbfA* pLMF03:*tbfA*	*R. anatipestifer* ATCC 1184*5, tonBA* mutant, *tonBB* mutant, *tbfA* mutant, pLMF03:*tbfA*, Cfx^*R*^	This study

**Plasmids**	**Genotype**	**Source or reference**

pOES	Suicide plasmid carrying EXpheS*, Amp^*R*^, Cfx^*R*^	Liu et al., 2018
pOES*:tonBA* up-down	*tonBA* up-down was cloned into pOES	This study
pOES*:tonBB* up-down	*tonBB* up-down was cloned into pOES	This study
pOES*:tbfA* up-down	*tbfA* up-down was cloned into pOES	This study
pLMF03	*B739_0921* promoter, *ori*ColE1, *ori* pRA0726, Amp^*R*^, Cfx^*R*^	Liu et al., 2016
pLMF03*:tonBA*	Plasmid pLMF03 containing *tonBA* insert	Liu et al., 2016
pLMF03*:tonBB*	Plasmid pLMF03 containing *tonBB* insert	Liu et al., 2016
pLMF03*:tbfA*	Plasmid pLMF03 containing *tbfA* insert	Liu et al., 2016

*Erm^*R*^ erythromycin resistance, Amp^*R*^ ampicillin resistance, Km^*R*^ kanamycin resistance, Cfx^*R*^ cefoxitin resistance.*

**TABLE 2:** Primers used in this study.

Primer	Organism	Sequence
*RA0C_1551* upP1	RA ATCC 11845	CTGAATCTCTTTTGGATAGTCTAGCCAATT
*RA0C_1551* downP2	RA ATCC 11845	TTTCTTCGTTTTTTATCATAATATTTAAATAAGAAAAC
*tonBA* upP1	RA ATCC 11845	CCGCTCGAGCGGAGAAAGGGCTTAGCAGAATAG
*tonBA* upP2	RA ATCC 11845	CTAAAATCCTTTTATTGATTTGGCTAAGTTTACTTTTCTTGTACGG
*tonBA* downP1	RA ATCC 11845	CCGTACAAGAAAAGTAAACTTAGCCAAATCAATAAAAGGATTTTAG
*tonBA* downP2	RA ATCC 11845	GGACTAGTCC GTTTAAGTCATTTAGCCTTCTAGC
*tonBB* upP1	RA ATCC 11845	GCTCTAGAGCGCCATCAAAGGCGATGGTAACACTAACTATCC
*tonBB* upP2	RA ATCC 11845	CTTGAACGAACTGGCTCTCCGCTCTGTCTTAGGTCATAAGCACCATAGGC
*tonBB* downP1	RA ATCC 11845	GCCTATGGTGCTTATGACCTAAGACAGAGCGGAGAGCCAGTTCGTTCAAG
*tonBB* downP2	RA ATCC 11845	CCGCTCGAGCGGCAATAAAGACCACAGCATCTCCCGC
*tbfA* upP1	RA ATCC 11845	CCGCTCGAGCGGCTATCTAGACATCAATGGTTCTATCGCTCAGCTAG
*tbfA* upP2	RA ATCC 11845	GGTCAAAAATTGTAATTATTTATTGTCATAAAATTTAAAATTAATAC
*tbfA* downP1	RA ATCC 11845	ATTTTAAATTTTATGACAATAAATAATTACAATTTTTGACCAATC
*tbfA* downP2	RA ATCC 11845	GGACTAGTCCAGCCTACCATCATACATTGTTAGAAGAAGTCCTTC
*Cfx* P1	pLMF03	CGGGGTACCTGACCCCGAAGCAGGGTTATGC
*Cfx* P2	pLMF03	GCTCTAGAGCAAAGCAAGTGCAGTTTAAGATTTTACTG
*EXpheS* P1	pLMF03:*pheS*	ACGCGTCGACATTTCAAAAATTTAACTTAAAACCACTG
*EXpheS* P2	pLMF03:*pheS*	GCTCTAGAGCCCTTTTTTTGTTACTTATAGCG
*16s rRNA* qRTP1	RA ATCC 11845	CGAAAGTGATAAGTTAGCCACCT
*16s rRNA* qRTP2	RA ATCC 11845	GCAGCACCTTGAAAATTGTCC
*dprA* qRTP1	RA ATCC 11845	TCCGATGTTTGAGGCAATTTG
*dprA* qRTP2	RA ATCC 11845	TGCAAGTTTGGTTAGCGAGGTAG
*comEC* qRTP1	RA ATCC 11845	CAATCCGAAATCTAACAGGCAAC
*comEC* qRTP2	RA ATCC 11845	CGAAGTGGCTTGGCACATATT
*comM* qRTP1	RA ATCC 11845	GTCGCCGCATCATACTATTTCC
*comM* qRTP2	RA ATCC 11845	ATCCTCCAAAGGTTGCCTCATA
*tonBA* qPCR P1	RA ATCC 11845	AAAGGAGGAAGTCGTAAGC
*tonBA* qPCR P2	RA ATCC 11845	TGAGGTTCTACAGGTGTAGG
*tonBB* qPCR P1	RA ATCC 11845	TGGTGCTTATGACCTAAGAC
*tonBB* qPCR P2	RA ATCC 11845	TCTACTTCTTGTTTAGGCGG
*tbfA* qPCR P1	RA ATCC 11845	ATGAGCTCTTATTTGCGGG
*tbfA* qPCR P2	RA ATCC 11845	CTCCAAATACAGCTACTCCTG
*RA0C_RS09540*qPCR P1	RA ATCC 11845	AGTACCTGCATCTACCTACG
*RA0C_RS09540*qPCR P2	RA ATCC 11845	GCATCATCAGCGATACTTCC
*RA0C_RS09840* qPCR P1	RA ATCC 11845	GAACTCACGAATATGCCAATACC
*RA0C_RS09840* qPCR P2	RA ATCC 11845	CCTATCCGTAACAGACCAACC

### Media and Growth Conditions

The frozen bacterial stocks of *R. anatipestifer* ATCC 11845 were cultured on sheep blood agar plates for 16–18 h at 37°C. Then, the cells from a single colony were inoculated to GC broth (GCB). GCB medium was prepared by supplementing 1 L of H_2_O with 1.5% peptone (Oxoid, China), 0.4% K_2_HPO_4_ (Sigma), 0.1% KH_2_PO_4_ (Sigma), and 0.5% NaCl (Sigma) plus 1% Kellogg’s supplements I and 0.1% Kellogg’s supplements II as described in a previous study (Liu et al., 2017b). Kellogg’s supplements I containing 40% glucose (Sigma), 1% L-glutamine (Sigma), and 0.002% vitamin B1; Kellogg’s supplements II containing 20 mM Fe (NO_3_)_3_. Kellogg’s supplements I was sterilized by a 0.45 μm filter, others were sterilized using an autoclave. GCB agar plates were prepared by GCB supplementation with 1.5% agar. *Escherichia coli* (*E. coli*) strains were routinely cultured in LB liquid medium or on LB agar plates at 37°C. Antibiotics were added at the following final concentrations: 1 μg/ml erythromycin (Erm), 1 μg/ml cefoxitin (Cfx), 20 μg/ml kanamycin (Kana), and 50 ng/m1 streptonigrin for *R. anatipestifer* and 100 μg/ml ampicillin (Amp) for *E. coli.*

### Preparation of Transforming DNA (tDNA)

Transforming DNA (tDNA) for natural transformation experiments was amplified from the strain *R. anatipestifer* ATCC 11845 Δ*RA0C_1551* using the primers *RA0C_1551* upP1 and *RA0C_1551* downP2. The tDNA contains the upstream of *RA0C_1551*, the Erm antibiotic resistance cassette, and the downstream of *RA0C_1551*. The strain *R. anatipestifer* ATCC11845Δ*RA0C_1551* was constructed by transformation-mediated recombination in our previous study (Liu et al., 2017b). The fragments were purified using the TianGEN Extract II Kit (TIANGEN, Beijing, China). The concentration of the fragments was measured by a NanoDrop 2000 spectrophotometer.

### Natural Transformation Assays

The standard natural transformation assay was performed as described previously (Liu et al., 2017b). Briefly, the bacteria were grown to the exponential phase (OD_600_ = 1.5–2.0) under aerobic conditions with shaking at 37°C. Then, the bacteria were harvested and resuspended to an OD_600_ of 1 in the GCB medium. Then, 0.3 ml of bacterial cells were transferred to sterilized tubes, and 1 μg of tDNA fragments was added. The transformation was allowed to proceed at 37°C for 1 h. Unabsorbed DNA was removed by washing, and then, the bacteria were plated on GCB plates containing 1 μg/ml Erm to obtain the transformants and on GCB plates to obtain the total viable bacteria, respectively. Transformation frequencies were calculated as the number of transformants divided by the total viable bacteria (Kristensen et al., 2012).

### The Effect of Different Components of GCB on Natural Transformation

The exponential phase *R. anatipestifer* ATCC 11845 cultures (OD_600_ = 1.5–2.0) were harvested, washed, and then resuspended to an OD_600_ of 1 in fresh GCB medium without one of the following: glucose, L-glutamine, vitamin B1, Fe (NO_3_)_3_, NaCl, phosphate (K_2_HPO_4_ and KH_2_PO_4_), or peptone. Following preincubation at 37°C for 1 h with shaking, the bacterial suspensions (0.3 ml) were used to perform the natural transformation in these nutrient-limited medium. In parallel, the GCB medium without peptone or phosphate was supplemented with different concentrations of peptone (0.5, 1 and 1.5%) or phosphate (0.1–0.5%). Then, bacteria in exponential growth were harvested by centrifugation, washed, and resuspended to an OD_600_ of 1 in these media. After preincubation for 1 h at 37°C with shaking, 0.3 ml of bacterial suspensions was used to perform the natural transformation in these media, respectively. In addition, sterile H_2_O was supplemented with 1.5% peptone, 0.5% phosphate (0.4% K_2_HPO_4_ and 0.1% KH_2_PO_4_), 0.5% NaCl, 0.4% glucose, 0.01% L-glutamine, 0.00002% VB_1_, 20 μM Fe (NO_3_)_3_, 1.5% peptone plus 0.5% phosphate, or 0.5% NaCl plus 0.5% phosphate, respectively. Then, the exponentially growing *R. anatipestifer* ATCC 11845 cells in GCB were collected by centrifugation, washed, and resuspended to an OD_600_ of 1 in these media. Following 1 h of preincubation, the bacterial suspensions (0.3 ml) were used to perform natural transformation in these media, respectively.

### Natural Transformation Under Iron-Limited Conditions

The bacteria were grown to an exponential phase in the GCB medium (OD_600_ = 1.5–2.0) and then resuspended to an OD_600_ of 1 in the GCB medium supplemented with 100 μM 2,2′-dipyridyl (Dip), which is able to chelate iron. In parallel, the bacterial suspensions (containing 100 μM Dip) were divided into five fractions, and 0, 2, 4, 20, and 120 μM Fe (NO_3_)_3_ were added, respectively. Following 1 h of preincubation, 0.3 ml of the bacterial suspension was used to perform natural transformation in these media, respectively.

### *In vitro* Growth Rate Determination

The *in vitro* growth rates of *R. anatipestifer* ATCC 11845 under the experimental conditions described above were determined at OD_600_ in the spectrophotometer (Eppendorf Biophotometer, Germany). Briefly, the overnight cultured bacterial cells were inoculated into 20 ml of the fresh medium at OD_600_ = 0.05 and incubated at 37°C with shaking at 180 rpm. The OD_600_ value was measured every 2 h for 14 h. Cultures were diluted to bring the OD600 at ∼0.5 when measured in 1.0 cm path length cuvettes.

### Construction of *TonB* Markerless Mutants and Complemented Strains in *R. anatipestifer* ATCC 11845

The knockout plasmids pOES:*tonBA*, pOES:*tonBB*, and pOES:*tbfA* were constructed using the primers listed in Table 2. The construction of *tonB* mutants was performed as described in a previous study (Liu et al., 2018). For complementation, the pLMF03 derivatives were transformed into relevant *R. anatipestifer* ATCC 11845 strains by conjugation as described previously (Liu et al., 2016).

### Sensitivity Assay Under Streptonigrin Stress

The overnight cultured bacterial cells were inoculated into fresh GCB liquid medium at an OD_600_ of 0.05. The cultures were incubated at 37°C with shaking (180 rpm) until they reached an OD_600_ = 1.5–2.0, and then, the bacteria were harvested by centrifugation, washed, and resuspended in PBS to be adjusted to an OD_600_ = 0.5. The total viable bacteria of 1 ml were counted by plating on the GCB plate. The final concentration of 50 ng/ml streptonigrin was added to 1 ml of the bacterial suspension, and the mixture was incubated at 37°C for 30 min. The incubated samples were washed and diluted serially to 10^–5^, 10^–6^, and 10^–7^ with PBS; 100 μl of each dilution was spread onto GCB agar plates, and the viable bacteria were counted after incubation overnight at 37°C. The survival rate was calculated by the number of surviving bacteria divided by the total number of viable bacteria.

### qRT-PCR

*R. anatipestifer* ATCC 11845 was grown in the GCB medium starting at OD_600_ = 0.05 at 37°C with shaking at 180 rpm. When the cultures reached the exponential growth phase (OD_600_ = 1.5–2.0), half of the bacteria were transferred to the GCB medium containing 100 μM Dip. After 1 h of incubation with shaking, the bacterial cells in GCB and GCB with 100 μM Dip were harvested for RNA extraction. Similarly, the exponentially bacterial cells were transferred to the GCB medium without phosphate, peptone, or phosphate plus peptone and incubated for 1 h at 37°C. Then, the cells were collected for RNA extraction. For the analysis of the transcription level of *tonBs*, the wild-type, *tonBA* mutant, *tonBB* mutant, and *tbfA* mutant strains were grown in the GCB medium until they reached the exponential growth phase (OD_600_ = 1.5–2.0) and were harvested for total RNA extraction. Total RNA extraction was performed using the RNeasy Protect Bacteria Mini Kit (QIAGEN, Cat. Number 74524) as described in a previous study (Liu et al., 2016). cDNA was synthesized from each RNA sample according to our recent study (Liu et al., 2017b). Real-time PCR assays were conducted using the primers listed in Table 2. Quantitative PCR was performed on samples in triplicate using the standard curve protocol in which the calibration curve was generated using serial fivefold dilutions of 100 ng of total cDNA. The RNA quantity was normalized using a probe specific for the 16S rRNA gene. Quantitative measurements were performed on biological samples in triplicate.

### Statistical Analysis

Statistical analyses were performed using the GraphPad Prism 6 (GraphPad software, CA, United States) for Windows. Statistical significance was evaluated using the Student’s *t*-test, one-way ANOVA, or two-way ANOVA. *p* < 0.05 represents statistically significant differences. Error bars in all figures represent the standard deviations of three independent experiments.

## Results

### Natural Transformation of *R. anatipestifer* ATCC 11845 Under Nutrient-Restricted Conditions

Since *R. anatipestifer* is naturally competent in all growth phases, and the transformation frequency is highest in the exponential growth phase (Liu et al., 2017b) and cell density do not play a significant role in competence development (data not shown), we suspected that the nutritional environment affects bacterial natural transformation. Next, we studied the effect of nutrient components in the GCB medium on the natural transformation of *R. anatipestifer* ATCC 11845, since the GCB medium promotes optimal competence and DNA uptake (Liu et al., 2017b, 2018; Liu M. et al., 2019).

**FIGURE 1:**
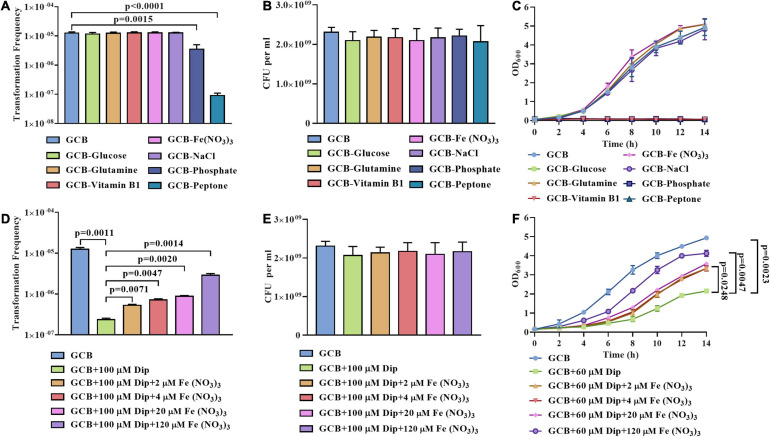
Natural transformation assay of *R. anatipestifer* ATCC 11845 in nutrient-limited GCB medium. **(A)** The natural transformation frequency of *R. anatipestifer* ATCC 11845 in the GCB medium and GCB medium without glucose, L-glutamine, vitamin B1, Fe (NO_3_)_3_, NaCl, phosphate, and peptone, respectively. The bacteria were grown to the exponential phase in the GCB medium, then, they were resuspended to an OD_600_ of 1 in fresh GCB medium and GCB medium without one of the following: glucose, L-glutamine, vitamin B1, Fe (NO_3_)_3_, NaCl, phosphate (K_2_HPO_4_ and KH_2_PO_4_), and peptone, respectively. Following preincubation at 37°C for 1 h with shaking, the bacterial suspensions (0.3 ml) were used to perform the natural transformation assay as described in section “Materials and Methods.” **(B)** The input bacterial titer of ∼2.5 × 10^9^ CFU were suspended in 1 ml GCB medium and GCB without glucose, L-glutamine, vitamin B1, Fe(NO_3_)_3_, NaCl, phosphate, and peptone, respectively. After 1 h of incubation at 37°C, the viability of *R. anatipestifer* ATCC 11845 was determined. The number of survival bacteria did not change significantly (*p* > 0.05). **(C)** The growth curve of *R. anatipestifer* ATCC 11845 in the GCB medium and GCB medium without glucose, L-glutamine, vitamin B1, Fe (NO_3_)_3_, NaCl, phosphate, and peptone, respectively. The bacteria were grown to the exponential phase in the GCB medium, then, they were resuspended in fresh GCB medium and GCB medium without one of the following: glucose, L-glutamine, vitamin B1, Fe (NO_3_)_3_, NaCl, phosphate (K_2_HPO_4_ and KH_2_PO_4_), and peptone, respectively, to an OD_600_ of 0.05. The bacteria were cultured at 37°C with shaking, and the OD_600_ was measured every 2 h for 14 h. **(D)** The effect of iron on the natural transformation frequency of *R. anatipestifer* ATCC 11845. The bacteria were grown to the exponential phase in the GCB medium, then, they were resuspended to an OD_600_ of 1 in fresh GCB medium, GCB medium containing 100 μM Dip, and GCB medium containing 100 μM Dip was supplemented with 2, 4, 20, and 120 μM Fe(NO_3_)_3_, respectively. Following preincubation at 37°C for 1 h with shaking, the bacterial suspensions (0.3 ml) were used to perform the natural transformation assay as described in section “Materials and Methods.” **(E)** The input bacterial titer of ∼2.5 × 109 CFU were suspended in 1 ml of GCB medium, GCB medium containing 100 μM Dip, GCB medium containing 100 μM Dip supplemented with 2, 4, 20, and 120 μM Fe(NO_3_)_3_, respectively. After 1 h of incubation at 37°C, the viability of *R. anatipestifer* ATCC 11845 was determined. The number of survival bacteria did not change significantly (*p* > 0.05). **(F)** The growth curve of *R. anatipestifer* ATCC 11845 in the GCB medium, GCB medium containing 60 μM Dip, and GCB containing 60 μM Dip supplemented with 2, 4, 20, and 120 μM Fe(NO_3_)_3_, respectively. The bacteria were grown to the exponential phase in the GCB medium, then, they were resuspended in fresh GCB medium, GCB medium containing 60 μM Dip, and GCB containing 60 μM Dip supplemented with 2, 4, 20, and 120 μM Fe(NO_3_)_3_, respectively. The bacteria were cultured at 37°C with shaking, and the OD_600_ was measured every 2 h for 14 h.

First, we subjected cells from exponential phase cultures to resuspension in the GCB medium without glucose, L-glutamine, vitamin B1, Fe (NO_3_)_3_, NaCl, phosphate, or peptone to perform natural transformation as described in section “Materials and Methods.” As shown in Figure 1A, no significant differences in the transformation frequency of *R. anatipestifer* ATCC 11845 were observed in the GCB medium without glucose, L-glutamine, vitamin B1, Fe(NO_3_)_3_, or NaCl. In contrast, the transformation frequency was significantly decreased when phosphate or peptone was removed (Figure 1A). To rule out the possibility of bacterial death, we also assessed the viability and growth in these nutrient-restricted media and found that *R. anatipestifer* ATCC 11845 survived well in these nutrient-restricted media during natural transformation assay (Figure 1B), but they did not grow in the GCB medium without vitamin B1, peptone, or phosphate (Figure 1B). Specifically, *R. anatipestifer* ATCC 11845 was observed to grow well in the GCB medium without Fe(NO_3_)_3_ (Figure 1C), suggesting that it was not an iron-limited medium. To create the iron-limited condition, the iron chelator 2,2’-dipyridyl (Dip) was added in the GCB medium without Fe(NO_3_)_3_. Thus, we compared the natural transformation frequency of *R. anatipestifer* ATCC 11845 in the GCB medium and GCB medium supplemented with 100 μM Dip as described in section “Materials and Methods.” As shown in Figure 1D, the transformation frequency decreased by approximately 100-fold when Dip was added. In addition to chelating Fe^2+^, Dip, as a divalent cation chelator, can also chelate other cations. To determine if the effect was caused by iron, we further performed natural transformation in GCB (100 μM Dip) supplemented with 0, 2, 4, 20, and 120 μM Fe (NO_3_)_3_, respectively. The result showed that the transformation frequency was gradually restored, suggesting that the natural transformation of *R. anatipestifer* ATCC 11845 requires iron. However, even though a high concentration of Fe (NO_3_)_3_ was added, the transformation frequency could not be completely restored to the original level. It was suggested that Dip may chelate other cations related to natural transformation (Figure 1D). We also measured the effect of Dip on the viability and growth of *R. anatipestifer* ATCC 11845, and the results showed that Dip did not affect bacterial survival during natural transformation assay (Figure 1E). However, the bacterial cells did not grow in the GCB medium supplemented with 100 μM Dip (data not shown). Thus, we chose to culture *R. anatipestifer* ATCC 11845 in the GCB medium containing 60 μM Dip and found that the growth of bacteria was significantly inhibited but gradually restored when Fe (NO_3_)_3_ was added (Figure 1F). These results indicated that phosphate, peptone, and iron affected the natural transformation of *R. anatipestifer* ATCC 11845 in the GCB medium. In contrast, depletion of vitamin B1, which is essential for the growth of *R. anatipestifer* ATCC 11845, did not have any effect on the natural transformation.

**FIGURE 2:**
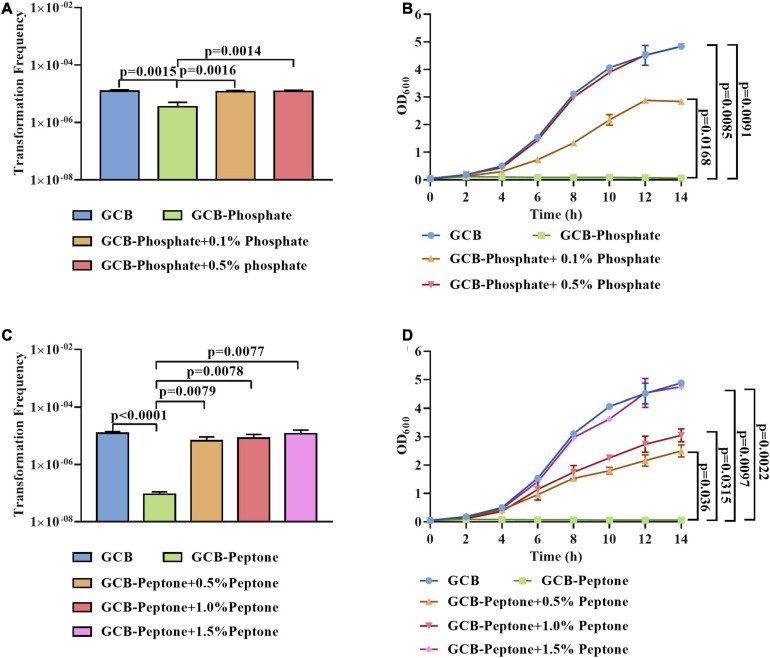
The effect of phosphate and peptone on the natural transformation of *R. anatipestifer* ATCC 11845, respectively. **(A)** The natural transformation frequency of *R. anatipestifer* ATCC 11845 in the GCB medium, GCB medium without phosphate, and GCB medium without phosphate supplemented with 0.1 and 0.5% phosphate, respectively. The bacteria were grown to the exponential phase in the GCB medium, then, they were resuspended to an OD_600_ of 1 in fresh GCB medium, GCB medium without phosphate, and GCB medium without phosphate supplemented with 0.1 and 0.5% phosphate, respectively. Following preincubation at 37°C for 1 h with shaking, the bacterial suspensions (0.3 ml) were used to perform the natural transformation assay as described in section “Materials and Methods.” **(B)** The growth curve of *R. anatipestifer* ATCC 11845 in GCB, GCB without phosphate, and GCB medium without phosphate supplemented with 0.1 and 0.5% phosphate, respectively. The bacteria were grown to the exponential phase in the GCB medium, then, they were resuspended in GCB, GCB without phosphate, and GCB medium without phosphate supplemented with 0.1 and 0.5% phosphate, respectively, to an OD_600_ of 0.05. The bacteria were cultured at 37°C with shaking, and the OD_600_ was measured every 2 h for 14 h. **(C)** The natural transformation frequency of *R. anatipestifer* ATCC 11845 in GCB, GCB without peptone, and GCB without peptone supplemented with 0.5,1 and 1.5% peptone, respectively. The bacteria were grown to the exponential phase in the GCB medium, then, they were resuspended to an OD_600_ of 1 in fresh GCB medium, GCB medium without peptone, and GCB without peptone supplemented with 0.5,1 and 1.5% peptone, respectively. Following preincubation at 37°C for 1 h with shaking, the bacterial suspensions (0.3 ml) were used to perform the natural transformation assay as described in section “Materials and Methods.” **(D)** The growth curve of *R. anatipestifer* ATCC 11845 in GCB, GCB without peptone, and GCB without peptone supplemented with 0.5,1, and 1.5% peptone, respectively. The bacteria were grown to the exponential phase in the GCB medium, then, they were resuspended in GCB, GCB without peptone, and GCB without peptone supplemented with 0.5,1 and 1.5% peptone, respectively, to an OD_600_ of 0.05. The bacteria were cultured at 37°C with shaking, and the OD_600_ was measured every 2 h for 14 h.

To further investigate the effect of phosphate and peptone on the natural transformation frequency of *R. anatipestifer* ATCC 11845, we transformed the tDNA into exponentially growing bacterial cells in the GCB medium depleted for or supplemented with different concentrations of phosphate or peptone, respectively. As shown in Figure 2A, the transformation ability of *R. anatipestifer* ATCC 11845 was gradually restored by the addition of 0.1 and 0.5% phosphate to the GCB medium without phosphate. To exclude the possibility that phosphate might affect the pH, we measured the pH value of the phosphate-free GCB medium and GCB medium supplemented with different concentrations of phosphate and found that the pH did not change significantly in these media (data not shown). These results implied that the effect of phosphate on the natural transformation was not due to changes in the pH. In addition, we also assessed the bacterial growth and the result showed that the growth of *R. anatipestifer* ATCC 11845 could be restored when phosphate was added, and when the concentration of phosphate was 0.5%, the growth could return to its original level (Figure 2B). Similarly, as shown in Figure 2C, the lowest concentration of peptone tested (0.5%) was able to restore the transformation ability of *R. anatipestifer* ATCC 11845. In addition, the growth was partially restored by adding 0.5 or 1% peptone and was completely restored by adding 1.5% peptone (Figure 2D).

We also measured whether iron, phosphate, and peptone have additive effects on the natural transformation of *R. anatipestifer* ATCC 11845. As shown in Supplementary Figure 1A, compared with the GCB medium without phosphate or peptone, the natural transformation frequency was decreased significantly when both phosphate and peptone were removed. Similarly, the natural transformation frequency was decreased significantly in the GCB medium without peptone when 100 μM Dip was added (Supplementary Figure 1B).

### The Natural Competence of *R. anatipestifer* ATCC 11845 in Sterile H_2_O Supplemented With a Single Component of the GCB Medium

**FIGURE 3:**
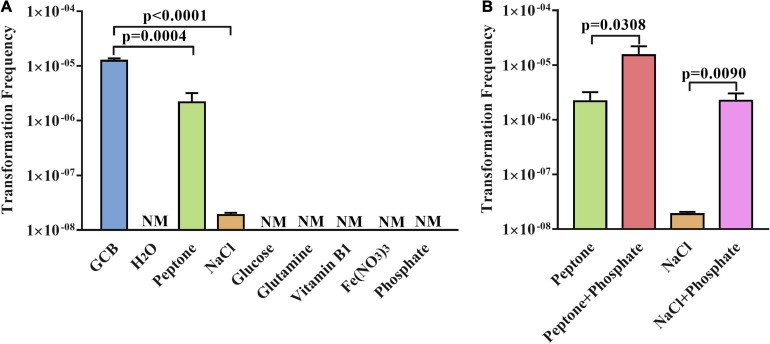
The natural transformation frequency of *R. anatipestifer* ATCC 11845 in sterile H_2_O supplemented with single components or double components of GCB. **(A)** The transformation assay of *R. anatipestifer* ATCC 11845 in sterile H_2_O_2_ supplemented by a single component of the GCB medium. The bacteria were grown to the exponential phase in the GCB medium; then, they were resuspended to an OD_600_ of 1 in fresh GCB medium, sterile H_2_O, sterile H_2_O supplemented with glucose, L-glutamine, vitamin B1, Fe (NO_3_)_3_, NaCl, phosphate, and peptone, respectively. Following preincubation at 37°C for 1 h with shaking, the bacterial suspensions (0.3 ml) were used to perform the natural transformation assay as described in section “Materials and Methods.” **(B)** Natural transformation assay of *R. anatipestifer* ATCC 11845 in sterile H_2_O containing peptone, NaCl, peptone and phosphate, NaCl and phosphate, respectively. The bacteria were grown to the exponential phase in the GCB medium, then, they were resuspended to an OD_600_ of 1 in sterile H_2_O supplemented with peptone, peptone and phosphate, NaCl, and NaCl and phosphate, respectively. Following preincubation at 37°C for 1 h with shaking, the bacterial suspensions (0.3 ml) were used to perform the natural transformation assay as described in section “Materials and Methods.” NM represents no transformant.

Next, to further verify the function of GCB components in natural transformation, we investigated whether a single component of GCB medium has an effect on the natural transformation of *R. anatipestifer* ATCC 11845. As described in section “Materials and Methods,” the transformation experiment was performed in sterile H_2_O and sterile H_2_O supplemented with the single component glucose, L-glutamine, vitamin B1, Fe (NO_3_)_3_, NaCl, phosphate, or peptone, respectively. The results showed that the natural competence of *R. anatipestifer* ATCC 11845 cannot occur in sterile H_2_O and sterile H_2_O supplemented with glucose, L-glutamine, vitamin B1, Fe (NO_3_)_3_, or phosphate, respectively. However, the transformants were detected after performing the natural transformation in sterile H_2_O supplemented with peptone or NaCl (Figure 3A), although only 20 ± 5 transformants per ∼10^9^ CFU were detected when the natural transformation was performed in sterile H_2_O supplemented with NaCl. In parallel, the bacterial cells survived well in these media during natural transformation (Supplementary Figure 2).

Although phosphate plays a crucial role in the natural transformation of *R. anatipestifer* ATCC 11845 in the GCB medium, we did not observe the occurrence of natural transformation in sterile H_2_O supplemented with phosphate (Figure 3A). This prompted us to further investigate the natural transformation of *R. anatipestifer* ATCC 11845 in sterile H_2_O containing peptone or NaCl by adding phosphate. The results showed that the transformation frequency increased significantly after the addition of phosphate in sterile H_2_O with peptone or NaCl (Figure 3B). As a control, the bacterial cells survived well in these media during the natural transformation but did not have any growth (data not shown).

### Transcription of the Natural Competence Genes *dprA*, *comEC*, *recA*, and *comM* Under Nutrition-Restricted Conditions

**FIGURE 4:**
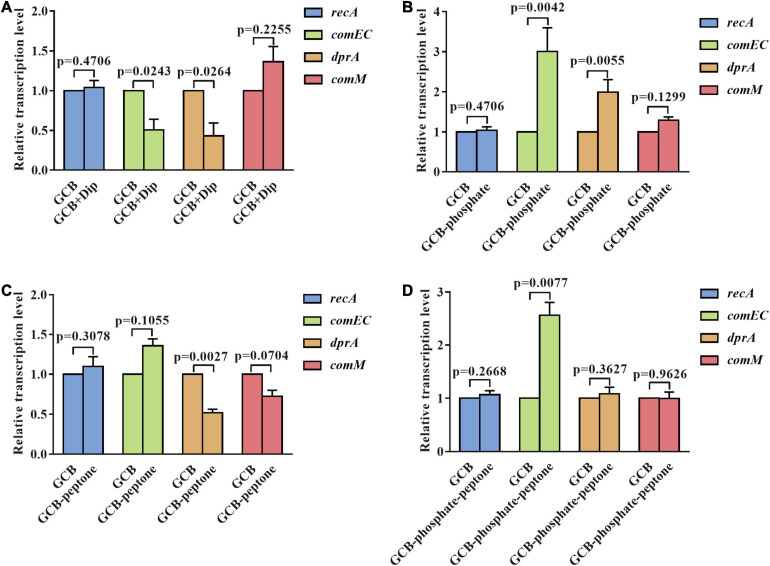
The transcriptional levels of *dprA, comEC, recA*, and *comM* in *R. anatipestifer* ATCC 11845 under different nutrient-limited conditions. **(A)** Quantitative real-time PCR analysis of the relative transcription of *dprA*, *comEC*, *recA*, and *comM* in *R. anatipestifer* ATCC 11845 in the GCB medium and GCB containing 100 μM Dip as described in section “Materials and Methods.” **(B)** Quantitative real-time PCR analysis of the relative transcription of *dprA*, *comEC*, *recA*, and *comM* in *R. anatipestifer* ATCC 11845 in GCB and GCB without phosphate as described in section “Materials and Methods.” **(C)** Quantitative real-time PCR analysis of the relative transcription of *dprA*, *comEC*, *recA*, and *comM* in *R. anatipestifer* ATCC 11845 in GCB and GCB without peptone as described in section “Materials and Methods.” **(D)** Quantitative real-time PCR analysis of the relative transcription of *dprA*, *comEC*, *recA*, and *comM* in *R. anatipestifer* ATCC 11845 in GCB and GCB without phosphate and peptone as described in section “Materials and Methods.” Fold changes were calculated by the _Δ Δ_ Ct method to consider the efficiency of the PCR for each target gene.

To further explore the mechanism affecting the natural transformation in *R. anatipestifer* ATCC 11845 in iron-limited GCB medium and in GCB medium without phosphate or peptone, we measured the transcription of conserved natural transformation genes in most competent bacteria, such as *dprA*, *comEC*, *recA*, and *comM*, which are involved in transporting ssDNA to the cytoplasm and facilitating RecA loading on internalized ssDNA and assisting homologous recombination (Johnston et al., 2014). The deletion of *dprA*, *comEC*, *recA*, and *comM* abolished the natural transformation of *R. anatipestifer* ATCC 11845 (Huang et al., 2019) (our unpublished data). As shown in Figure 4A, the transcription of *dprA* and *comEC* was significantly downregulated (twofold) under iron-limited conditions, whereas that of *recA* and *comM* was not changed significantly under the same conditions, indicating that the decreased transformation frequency of *R. anatipestifer* ATCC 11845 under iron-limited conditions could be related to lower *dprA* and *comEC* expression. Interestingly, the levels of *dprA* and *comEC* transcripts in the GCB medium without phosphate were observed to be increased by two and threefold, respectively, and those of *recA* and *comM* had almost no change (Figure 4B). This result suggested that the effect of phosphate on natural transformation was not caused by changes in *dprA*, *comEC*, *recA*, or *comM* transcription levels. Moreover, in the GCB medium without peptone, only the transcription of *dprA* was significantly downregulated (twofold), whereas that of *comEC*, *recA*, and *comM* showed no statistically significant changes (Figure 4C). As evidence, the qRT-PCR results showed that the transcription of *comEC* was upregulated (twofold), whereas that of *dprA*, *recA*, and *comM* were not significantly different in the GCB medium without phosphate and peptone (Figure 4D). These findings suggested that iron, phosphate, and peptone may act through different mechanisms or at different stages of the natural transformation process.

### The Effect of TonB on the Natural Transformation of *R. anatipestifer* ATCC 11845

**FIGURE 5:**
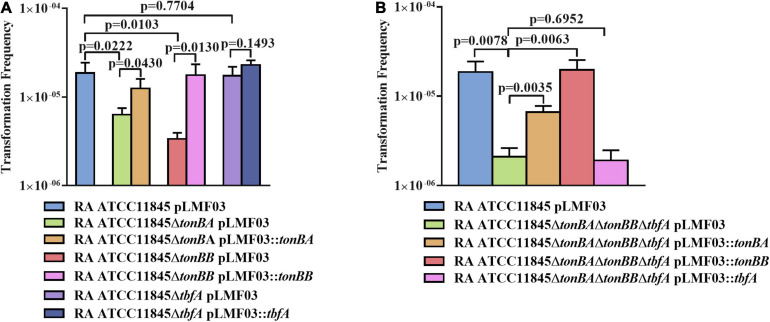
The effect of *tonB* mutant on the natural transformation of *R. anatipestifer* ATCC 11845 in GCB. **(A)** The natural transformation frequency of the *tonBA*, *tonBB*, or *tbfA* mutants of *R. anatipestifer* ATCC 11845 in the GCB medium. **(B)** The natural transformation frequency of the *tonBA–tonBB–tbfA* triple mutant of *R. anatipestifer* ATCC 11845 and the single TonB complementation strain in the GCB medium. Natural transformation assay was performed using the standard method as described in section “Materials and Methods.”

Since TonB of gram-negative bacteria is important for nutrient transportation, including iron (Noinaj et al., 2010), we further investigated whether TonB was involved in the natural transformation of *R. anatipestifer* ATCC 11845. In a previous study, it was shown that both TonBA and TonBB, but not TbfA, are required for hemin uptake in *R. anatipestifer* (Liao et al., 2015; Liu et al., 2016). As shown in Figure 5A, compared with the wild type, the natural transformation frequency was decreased significantly in the *tonBA* and *tonBB* mutants; however, the *tbfA* mutation did not have any effect on the natural transformation of *R. anatipestifer* ATCC 11845. Particularly, it was observed that the *tonBB* mutant inhibited the natural transformation more severely than the *tonBA* mutant. Similarly, the *tonBA–tonBB–tbfA* triple mutant of *R. anatipestifer* ATCC 11845 exhibited a decreased natural transformation frequency than the single mutants, and the complementation of TonBA or TonBB, but not TbfA, was able to partially restore the natural transformation (Figure 5B). As a control, it was shown that *tonB* single mutant did not have any effect on the survival of the bacteria in the GCB medium during the natural transformation (Supplementary Figure 3).

**FIGURE 6:**
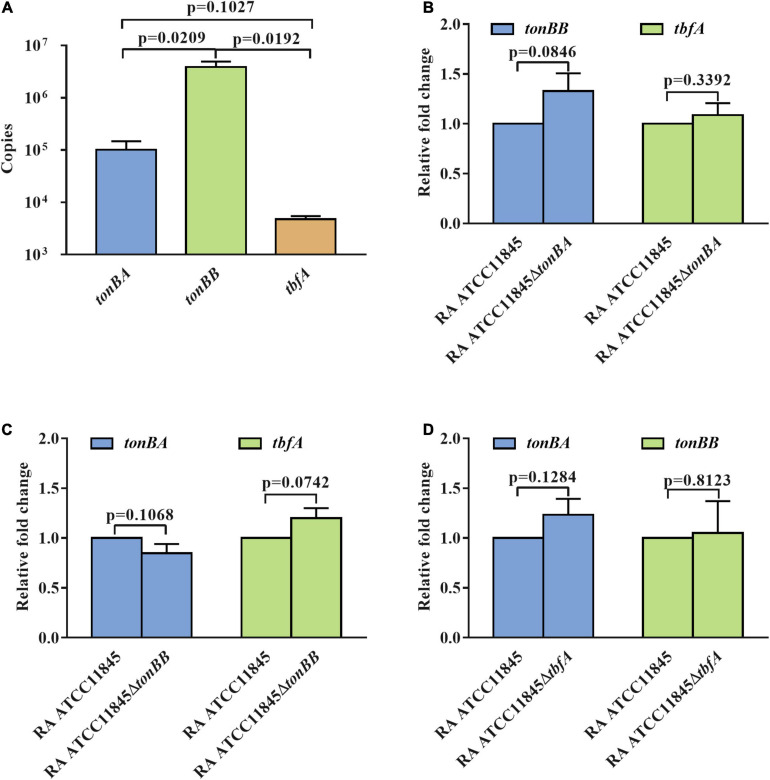
The transcription level of the three *tonBs* in *R. anatipestifer* ATCC 11845 and its derivative strains. **(A)** The transcriptional copies number of the three *tonBs* in *R. anatipestifer* ATCC 11845. The standard curves of gene copies number and *Ct* values were established by amplifying *tonBA*, *tonBB*, and *tbfA* from plasmid pLMF03:*tonBA*, pLMF03*:tonBB*, and pLMF03*:tbfA*, respectively, using quantitative real-time PCR. Subsequently, the *Ct* values of three *tonBs* in *R. anatipestifer* ATCC 11845 were measured by amplifying *tonBA*, *tonBB*, and *tbfA* from cDNA of *R. anatipestifer* ATCC 11845. Finally, the transcriptional copies numbers were calculated according to the constructed standard curves. **(B)** The relative transcription level of the *tonBB* and *tbfA* in the *tonBA* mutant. **(C)** The relative transcription level of the *tonBA* and *tbfA* in the *tonBB* mutant. **(D)** The relative transcription level of the *tonBA* and *tonBB* in the *tbfA* mutant. The *R. anatipestifer* ATCC 11845, *tonBA* mutant, *tonBB* mutant, and *tbfA* mutant were grown in the GCB medium, respectively, until reaching the exponential growth phase and harvested for total RNA extraction and real-time quantitative PCR as described in section “Materials and Methods.” Each experiment consisted of three biological replicate samples with three technical replicates each.

Previous studies have established that the expression of the *tonB* genes of *R. anatipestifer* is not regulated by iron availability (Liu et al., 2017a). We then determined whether TonBB is more important or highly expressed than the other two TonBs. The transcription of *tonBA*, *tonBB*, and *tbfA* in *R. anatipestifer* ATCC 11845 were measured and the results showed that *tonBB* had the highest transcriptional level, and *tbfA* had the lowest transcriptional level (Figure 6A). To identify whether the *tonB* mutants influence the transcription of the other two *tonBs*, we further measured the transcription level of the three *tonBs* in each *tonB* single mutant and found that the *tonB* single mutants did not have any effect on the transcription of the other two *tonBs* (Figures 6B–D). It is suspected that TonBA and TonBB, but not TbfA, are involved in the natural transformation of *R. anatipestifer* ATCC 11845.

### The *tonBB* Mutant, but Not the *tonBA* Mutant, Damages the Iron Uptake of *R. anatipestifer* ATCC 11845

**FIGURE 7:**
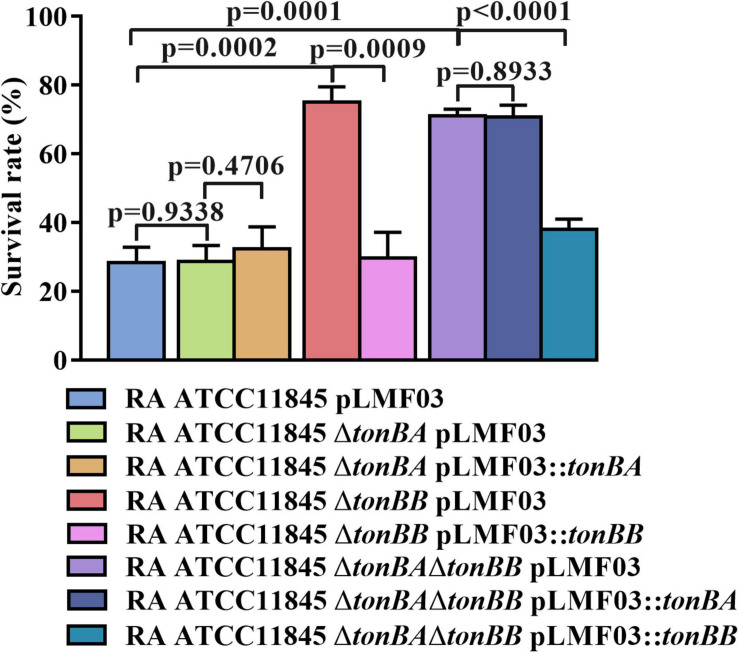
The survival rate of *R. anatipestifer* ATCC11845 and its derivative strains when they were treated by streptonigrin. The bacteria were grown in the GCB medium until the exponential growth phase, then, harvested and resuspended in PBS to an OD_600_ = 0.5. The final concentration of 50 ng/ml Streptonigrin was added to 1 ml of the bacterial suspension, and the mixture was incubated at 37°C for 30 min. The survival rate was calculated as described in section “Materials and Methods.”

TonBA and TonBB are involved in the natural transformation in the GCB medium, which can be reasoned by the deletion of *tonBA* or *tonBB* decreased the intracellular iron level in *R. anatipestifer* ATCC 11845. To verify this hypothesis, the sensitivity of the strains to streptonigrin, which exhibits an enhanced bacterial killing in the presence of iron (White and Yeowell, 1982), was compared. The results showed that *R. anatipestifer* ATCC11845 *ΔtonBA* did not exhibit a significant difference in survivability compared to the wild-type strain when treated with 50 g/ml of streptonigrin (Figure 7). However, the survival rate of *R. anatipestifer* ATCC11845 *ΔtonBB* was significantly higher than that of the wild-type strain when treated with 50 ng/ml streptonigrin (Figure 7). The survival rate of the *tonBAtonBB* double mutant did not change significantly compared to that of the *tonBB* single mutant when treated with 50 ng/ml streptonigrin (Figure 7). These results indicated that the *R. anatipestifer tonBB* mutant, but not the *tonBA* mutant, is defective in iron uptake.

If the concentration of intracellular iron is decreased, the putative iron uptake genes will be upregulated. Thus, we further measured the transcript levels of *RA0C_RS09540* and *RA0C_RS09840* in *R. anatipestifer* ATCC11845 *ΔtonBA* and *R. anatipestifer* ATCC11845 *ΔtonBB*, respectively, since their homologs were upregulated in *R. anatipestifer* CH-1 under iron-limited conditions (Liu et al., 2017b; Huang M. et al., 2021). As shown in Supplementary Figure 4A, compared to that of the wild type, *RA0C_RS09540* and *RA0C_RS09840* had higher transcript levels in *R. anatipestifer* ATCC11845 *ΔtonBB*, but not in *R. anatipestifer* ATCC11845 *ΔtonBA*, when the bacteria were grown in the GCB medium. As a control, the transcripts of *RA0C_RS09540* and *RA0C_RS09840* in the wild type were higher in an iron-limited medium than in iron-rich medium (Supplementary Figure 4B). Taken together, these results suggest that the *tonBB* mutant, but not the *tonBA* mutant, is defective in the iron uptake in *R. anatipestifer* ATCC11845 when grown in the GCB medium.

## Discussion

Natural transformation is a major mechanism of horizontal gene transfer in bacteria (Thomas and Nielsen, 2005). It has been shown that environmental cues play a crucial role in the occurrence of natural transformation (Seitz and Blokesch, 2013). As a newly identified natural competent bacterium, the factors that affect the natural transformation of *R. anatipestifer* are largely unknown. Here, we systematically examined the effects of nutrient factors on the natural transformation of *R. anatipestifer* ATCC 11845.

In a previous study, it was shown that the natural transformation of *R. anatipestifer* ATCC 11845 was constitutive and had a high transformation frequency in the exponential growth phase (Liu et al., 2017b). Thus, we hypothesize that the nutrition condition may affect the natural transformation of *R. anatipestifer*ATCC11845. In this study, it was shown that the transformation frequencies were decreased significantly with the removal of phosphate or peptone and were gradually restored by the addition of phosphate or peptone, suggesting that phosphate and peptone affected the natural transformation of *R. anatipestifer* ATCC 11845. Interestingly, we found that the natural competence of *R. anatipestifer* ATCC 11845 could occur in sterile H_2_O supplemented with peptone or NaCl, respectively, but not in phosphate. This result suggested that peptone and NaCl are able to induce the natural competence of *R. anatipestifer* ATCC 11845, while phosphate can enhance the natural transformation frequency but is not enough to initiate it. We further observed that the natural transformation of *R. anatipestifer* ATCC11845 also occurred in H_2_O supplemented by NaHCO*3*, but not KCl (data not shown), speculating that Na^+^ has positive charges that neutralize the negativity of the phosphate backbone of the DNA to be transformed. Since peptone is a complex mixture and its nutritive source is largely dependent on the amino acid content, we speculated that peptone supplies amino acids for protein synthesis in cells that need to make the transformation machinery.

Iron is an essential element for the growth and survival of most bacteria since it functions in cellular processes such as respiration, oxidative stress resistance, and DNA synthesis (Andrews et al., 2003; Huang M. et al., 2021). In our study, the natural transformation was inhibited by adding 100 μM Dip, and the transformation frequency was restored gradually by the supplementation with Fe(NO_3_)_3_. Dip is reported to be a chelator of divalent cations, which has been widely used in many studies to foster iron starvation (Lu et al., 2013; Yan et al., 2013; Karash and Kwon, 2018). It was suggested that the natural transformation of *R. anatipestifer* ATCC11845 could be affected by iron, implying that energy generation, as well as nucleotide synthesis, are important for the process of natural transformation. Notably, even though the high concentration of Fe(NO_3_)_3_ was added, the transformation ability cannot be completely restored to the original level. Our result was speculated that in addition to iron, the incorporation of Dip in the GCB medium may affect other divalent cation related to natural transformation. It was found that the member of *Flavobacteriaceae*, *R. columbina* was naturally competent and Mg^2+^, Zn^2+^, and Mn^2+^ promote the natural transformation frequency (Huang L. et al., 2021). Vitamin B1 is essential to nearly all cellular life, as it serves as a critical cofactor for many enzymes involved in carbohydrate metabolism (Sannino et al., 2018). In our study, we observed that *R. anatipestifer* ATCC 11845 could not grow in the GCB medium without vitamin B1, but the transformation frequency did not have any effect, suggesting that the natural transformability of *R. anatipestifer* may not be related to its growth.

Most gram-negative bacteria depend on the TonB complex to transport hemin and iron (Noinaj et al., 2010). Our previous studies showed that TonB proteins of *R. anatipestifer* are required for iron and hemin uptake (Liao et al., 2015; Liu et al., 2016, 2021). This prompted us to further determine whether TonB of *R. anatipestifer* ATCC 11845 was involved in the natural transformation. The results showed that the *tonBA* or *tonBB* mutant decreased the natural transformation frequency of *R. anatipestifer* ATCC 11845 in the GCB medium. Moreover, compared with the *tonBA* mutant, the *tonBB* mutant decreased the natural transformation frequency is more significant. Streptonigrin is an antibiotic exhibiting iron-dependent bactericidal activity and has been used as an indirect approach to estimate free iron content in bacterial cells (Ganguly et al., 2018). In this study, we used streptonigrin sensitivity assay to evaluate the intracellular iron level of *tonB* mutants and found that the *tonBB* mutant, but not the *tonBA* mutant, was defective in the iron uptake in the GCB medium, speculating that the loss of *tonBB* decreased the natural transformation frequency since it was damaged in iron utilization. Recently, it was reported that the TonB complex was required for the protein secretion in *Myxococcus xanthus* (Gomez-Santos et al., 2019). We speculated that the *tonBA* mutant may be damaged in the nutrient transportation related to natural transformation, such as peptone or phosphate, but this requires further study.

In summary, our study showed that peptone, phosphate, and iron influenced the transformation frequency of *R. anatipestifer* ATCC 11845 through a mutually independent pathway. In addition, our study also showed that TonBA and TonBB are involved in the natural transformation of *R. anatipestifer*. These observations provide further insights for understanding the natural transformation of *R. anatipestifer*.

## Data Availability Statement

The original contributions presented in the study are included in the article/Supplementary Material, further inquiries can be directed to the corresponding author/s.

## Author Contributions

LZ, ML, and AC conceived and designed the research. LZ, LH, and MH performed the experiments and wrote the manuscript. MeW, DZ, MiW, RJ, SC, and XZ participated in the experiments. QY, YW, SZ, and JH contributed the analysis tools. XO, SM, QG, and BT supervised the studies and corrected the manuscripts. All authors read and approved the final manuscript.

## Conflict of Interest

The authors declare that the research was conducted in the absence of any commercial or financial relationships that could be construed as a potential conflict of interest.

## Publisher’s Note

All claims expressed in this article are solely those of the authors and do not necessarily represent those of their affiliated organizations, or those of the publisher, the editors and the reviewers. Any product that may be evaluated in this article, or claim that may be made by its manufacturer, is not guaranteed or endorsed by the publisher.
