# Supplementary material for: Effect of Nutritional Determinants and TonB on the Natural Transformation of Riemerella anatipestifer
Source: Front Microbiol. 2021 Aug 10;12:644868. doi: 10.3389/fmicb.2021.644868 (PMC8383284; doi:10.3389/fmicb.2021.644868)
Supplement: Supplementary file 1 [file Table_1.docx]

**SUPPORTING INFORMATION**

**Figure S1. The additive effects of iron, phosphate and peptone on the natural transformation of *R. anatipestifer* ATCC 11845.**


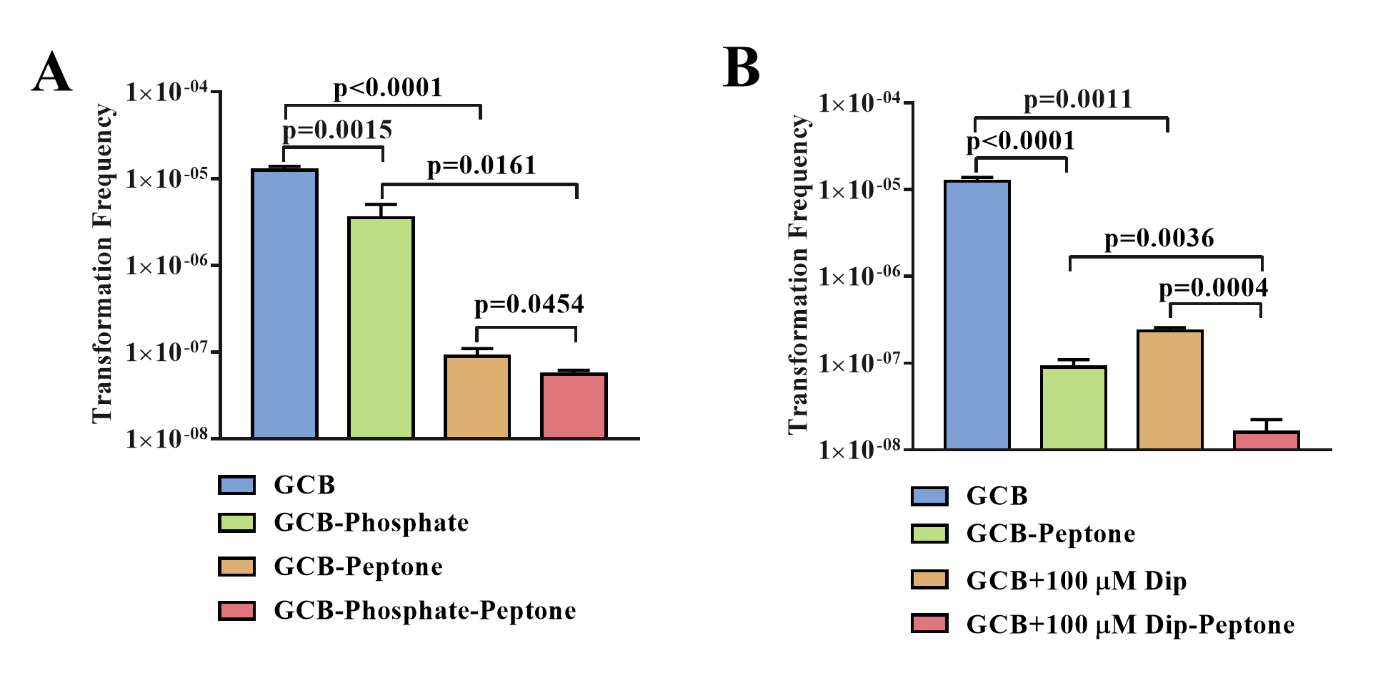


**Figure S1 The additive effects of iron, phosphate and peptone on the natural transformation of *R. anatipestifer* ATCC 11845.** (A) The bacteria were grown to exponential phase in GCB and then they were resuspended to an OD_600_ of 1 in GCB medium, GCB medium without phosphate, GCB medium without peptone, GCB medium without both phosphate and peptone, respectively. Following preincubation at 37°C for 1 h with shaking, the bacterial suspensions (0.3 ml) were used to perform the natural transformation assay as described in “Materials and methods”. (B) The bacteria were grown to exponential phase in GCB and then they were resuspended to an OD_600_ of 1 in GCB medium, GCB medium without peptone, GCB medium supplemented by 100 μM Dip, GCB medium without peptone and supplemented by 100 μM Dip, respectively. Following preincubation at 37°C for 1 h with shaking, the bacterial suspensions (0.3 ml) were used to perform the natural transformation assay as described in “Materials and methods”. The data were analyzed using Student’s t-test. Error bars represent the standard deviation obtained from three independent measurements.

**Figure S2. The survival of *R. anatipestifer* ATCC 11845 in the sterile H_2_O supplemented with the single component of GCB medium during natural transformation.**

**

**

**Figure S2. The survival of *R. anatipestifer* ATCC 11845 in the sterile H_2_O supplemented with the single component of GCB medium during natural transformation.** The input bacterial titre ~2.5×10^9^CFU were suspended in 1 ml in fresh GCB medium, sterile H_2_O, sterile H_2_O supplemented with glucose, L-glutamine, vitamin B1, Fe (NO_3_)_3_, NaCl, phosphate and peptone, respectively. After 1 h incubation at 37°C, the viability of *R. anatipestifer* ATCC 11845 was determined. The data were analyzed using Student’s t-test. The number of survival bacteria did not change significantly(P>0.05). Error bars indicate standard deviation of three independent measurements.

**Figure S3. The viability of *R. anatipestifer* ATCC 11845 and *tonB* mutants in GCB medium**


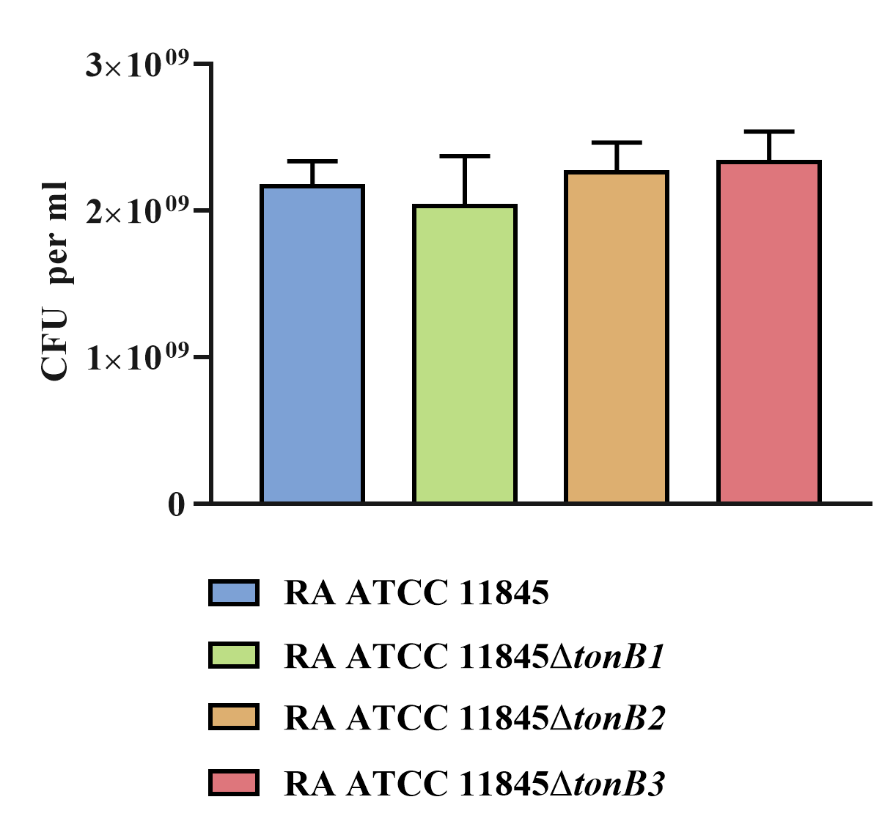


**Figure S3. The viability of *R. anatipestifer* ATCC 11845 and *tonB* mutant in GCB during natural transformation assay.** The number of survival bacteria did not change significantly(P>0.05).

**Figure S4 The relative transcription level of *RA0C_RS09540* and *RA0C_RS09840* in *R. anatipestifer* ATCC11845 and its derivative strains in GCB or iron limited GCB medium.**

**

**

**Figure S4. The relative transcription level of *RA0C_RS09540* and *RA0C_RS09840* in *R. anatipestifer* ATCC11845 and its derivative strains in GCB or iron limited GCB medium.** (A) The relative transcription level of *RA0C_RS09540* and *RA0C_RS09840* in *R. anatipestifer* ATCC11845, *R. anatipestifer* ATCC11845 ∆*tonBA* and *R. anatipestifer* ATCC11845 ∆*tonBB*, respectively. (B) The relative transcription level of *RA0C_RS09540* and *RA0C_RS09840* in *R. anatipestifer* ATCC11845 in GCB and GCB containing 100 μM Dip. The quantitative real-time PCR as described in “Materials and methods”. Each experiment consisted of three biological replicate samples with three technical replicates each. The data were analyzed using Student’s t-test. The error bars represent the standard deviation of three independent experiments.
